# Functional analyses of ancestral thioredoxins provide insights into their evolutionary history

**DOI:** 10.1074/jbc.RA119.009718

**Published:** 2019-07-31

**Authors:** Silvia Napolitano, Robin J. Reber, Marina Rubini, Rudi Glockshuber

**Affiliations:** ‡ Institute of Molecular Biology and Biophysics, Department of Biology, Swiss Federal Institute of Technology Zurich, Otto-Stern-Weg 5, CH-8093 Zurich, Switzerland; § School of Chemistry, University College Dublin, Belfield, Dublin 4, Ireland

**Keywords:** molecular evolution, thioredoxin, thioredoxin reductase, oxidative stress, electron transfer, methionine sulfoxide reductase, phylogenetic reconstruction, redox biology, redox homeostasis

## Abstract

Thioredoxin (Trx) is a conserved, cytosolic reductase in all known organisms. The enzyme receives two electrons from NADPH via thioredoxin reductase (TrxR) and passes them on to multiple cellular reductases via disulfide exchange. Despite the ubiquity of thioredoxins in all taxa, little is known about the functions of resurrected ancestral thioredoxins in the context of a modern mesophilic organism. Here, we report on functional *in vitro* and *in vivo* analyses of seven resurrected Precambrian thioredoxins, dating back 1–4 billion years, in the *Escherichia coli* cytoplasm. Using synthetic gene constructs for recombinant expression of the ancestral enzymes, along with thermodynamic and kinetic assays, we show that all ancestral thioredoxins, as today's thioredoxins, exhibit strongly reducing redox potentials, suggesting that thioredoxins served as catalysts of cellular reduction reactions from the beginning of evolution, even before the oxygen catastrophe. A detailed, quantitative characterization of their interactions with the electron donor TrxR from *Escherichia coli* and the electron acceptor methionine sulfoxide reductase, also from *E. coli*, strongly hinted that thioredoxins and thioredoxin reductases co-evolved and that the promiscuity of thioredoxins toward downstream electron acceptors was maintained during evolution. In summary, our findings suggest that thioredoxins evolved high specificity for their sole electron donor TrxR while maintaining promiscuity to their multiple electron acceptors.

## Introduction

**Figure 1.**
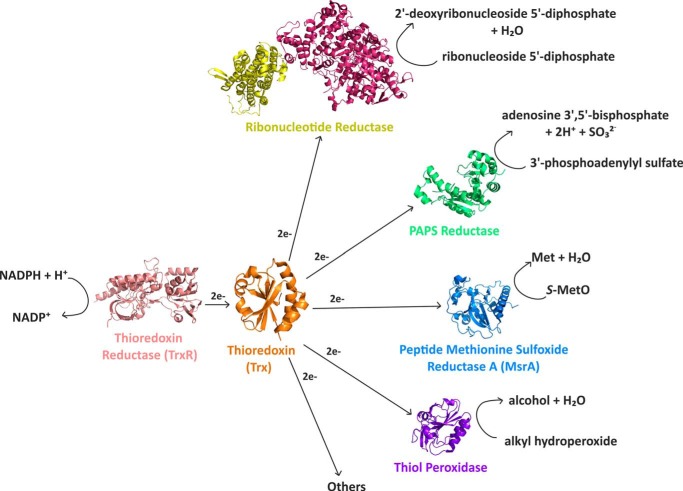
**NADPH-dependent reductive pathways catalyzed by thioredoxin in the *E. coli* cytosol.** Abbreviations used are as follows: thioredoxin reductase (*TrxR*), PDB code 1f6m (39); thioredoxin (*Trx*), PDB code 2trx (57); methionine sulfoxide reductase (*MsrA*), PDB code 1ff3 (58); PAPS reductase, PDB code 1sur (59); thiol peroxidase, PDB code 3hvs (60); ribonucleotide reductase, PDB code 3uus (61). Thioredoxin reductase, PAPS reductase, and thiol peroxidase are homodimers. For clarity, only cartoon representations of the respective monomers are shown. Ribonucleotide reductase is an α_2_β_2_ heterotetramer, of which only a αβ heterodimer is shown.

The phylogenetic reconstruction of ancestral enzymes dating back 4 billion years is one of the most fascinating areas in protein biochemistry. The structural, biophysical, and functional characterization of resurrected ancestral enzymes and their interactions provide important insights into the evolution of protein folding and stability, protein function, the metabolism of Precambrian organisms, and the conditions under which they existed (1–3). Among the protein families of which ancestral members have been reconstructed and characterized, the thioredoxin family is one of the best-studied examples. Thioredoxin (Trx)^2^ is a cytoplasmic 12-kDa oxidoreductase ubiquitously present in all domains of life (4). The conserved Trx fold consists of a single domain of about 110 residues forming a central, four-stranded β-sheet surrounded by three α-helices (5). The active site of thioredoxins consists of a catalytic cysteine pair within a characteristic Cys-Xaa-Xaa-Cys motif (where Xaa is any amino acid) at the N terminus of the first α-helix (4, 6, 7). All known cytoplasmic thioredoxins act as reductases in the oxidative stress response and NADPH-dependent reductive pathways (8, 9). Specifically, thioredoxins receive electrons from the NADPH-dependent flavoenzyme thioredoxin reductase (TrxR) via disulfide exchange and pass them on to multiple substrate enzymes with catalytic cysteine pairs, including ribonucleotide reductase (10), methionine sulfoxide reductase (11), phosphoadenosine phosphosulfate (PAPS) reductase (12), peroxidoredoxin (13), and the transmembrane reductase DsbD (Fig. 1) (14).

Previous studies on Precambrian thioredoxins focused on seven resurrected ancestral members dating between 1.4 and 4 billion years, namely the variants Last Bacterial Common Ancestor (LBCA), Last Archaeal Common Ancestor (LACA), Archaeal–Eukaryotic Common Ancestor (AECA), Last Eukaryotic Common Ancestor (LECA), Last Animalia and Fungi Common Ancestor (LAFCA), Last Common Ancestor of the Cyanobacterial, *Deinococcus,* and *Thermus* group (LPBCA), and Last γ-Proteobacteria Common Ancestor (LGPCA) (15). These ancestral thioredoxins share between 36% (LAFCA) and 84% (LGPCA) sequence identity with *Escherichia coli* Trx (ecTrx) (Table S1 and Fig. S1). Their primary structures had been predicted by phylogenetic sequence reconstruction, assuming that the root of the Trx sequence tree lies between bacteria and the common ancestor of archaea and eukaryotes, and by applying statistical methods based on maximum likelihood (15). The crystal structures of all seven ancestral thioredoxins were determined and proved to possess the same overall fold as ecTrx, the prototype of the thioredoxin family (16). Notably, in agreement with the hypothesis that life originated under extreme conditions and that Precambrian organisms grew at high temperatures (17), the resurrected ancestral thioredoxins showed extraordinary thermal stability compared with thioredoxins from modern mesophilic organisms, and they exhibited melting temperatures up to 123 °C (15). The ancestral thioredoxins also showed significantly higher free energies of folding at strongly acidic pH (pH 2.0) compared with *E. coli* Trx (ecTrx), which primarily originated from drastically slower unfolding rates (18). Further studies identified residues in the Trx fold with which the increased stabilities of ancestral thioredoxins correlate, allowing the prediction of stabilizing mutations (2, 19–22).

Despite these important insights into the mechanisms underlying the evolution of thermostability in the thioredoxin fold, comparably little is known about the *in vivo* function of resurrected ancestral thioredoxins in the context of a modern mesophilic organism. The only available report is a study by Delgado *et al.* (23), who tested the ability of the ancestral thioredoxins to complement Trx deficiency in a thioredoxin deletion (Trx^−^) mutant of *E. coli* in rich medium where Trx is not essential. In this nonstringent experimental setup, all ancestral Trx variants showed partial complementation of Trx deficiency, and growth curves demonstrated that doubling times decreased with increasing sequence identity between the ancestral thioredoxins and ecTrx. In addition, the ancestral thioredoxins were tested for their ability to replace ecTrx as a subunit of the T7 phage DNA polymerase and to enable T7 propagation. The results showed that only the Trx variant most closely related to ecTrx, LGPCA, was capable of restoring T7 replication in the *E. coli* Trx^−^ mutant. Notably, this study showed that ancestral thioredoxins can be used for engineering phage-resistant bacterial strains. Finally, the only available *in vitro* study in which the ancestral thioredoxins were functionally characterized showed that all ancestral enzymes were capable of reducing disulfide bonds in non-natural model substrates, including the disulfide in the mechanically unfolded immunoglobulin-like domain I27 and the disulfide bonds of insulin (15).

Here, we provide the first quantitative characterization of ancestral thioredoxins in their natural function as catalysts of NADPH-dependent reduction reactions in the context of today's *E. coli* cytoplasm. Specifically, we measured how efficiently the ancestral Trx variants receive electrons from *E. coli* thioredoxin reductase (TrxR), and how efficiently they pass electrons on to one of the natural downstream substrates, peptide methionine sulfoxide reductase A (MsrA). The functional analysis of the ancestral thioredoxins in the reconstituted electron transport pathway from NADPH via TrxR, Trx, and MsrA to methionine sulfoxide *in vitro* and their *in vivo* function in the same electron transport chain under selective pressure indicated that thioredoxins and thioredoxin reductases co-evolved, and that the ability of thioredoxins to efficiently reduce multiple natural and non-natural substrates has been preserved throughout evolution.

## Results

### Redox properties of the ancestral thioredoxins

**Figure 2.**
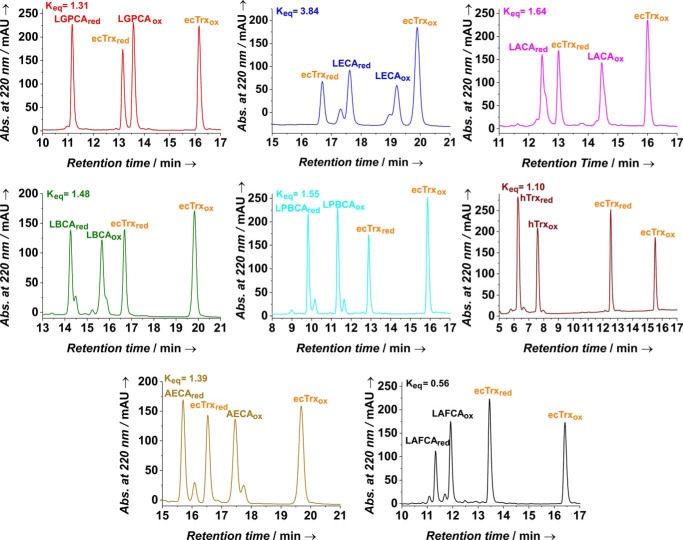
**Redox equilibria between ecTrx and the individual ancestral thioredoxins at pH 7.0 and 25 °C.** Reduced ecTrx was mixed with an equimolar amount of the oxidized form of the respective ancestral Trx. After attainment of equilibrium, the reactions were quenched with acid, and all redox forms were separated in a single run by reversed-phase HPLC. Peak areas were converted to concentrations, from which redox equilibrium constants (*K*_eq_) and redox potential (*E*′_0_) (Table 1) were calculated according to Equations 1 and 2 (see under “Experimental procedures”), respectively. The double peaks observed for both redox forms of the Trx variants AECA, LPBCA, LAFCA, and LECA result from incomplete intracellular cleavage of the N-terminal methionine during expression of these variants (see Fig. S3 and Table S2). To confirm that equilibrium was attained, reduced ecTrx and the oxidized ancestral variants were also mixed at different molar ratios (Fig. S2). The obtained *K*_eq_ resulted to be independent of the initial mixing ratio, proving that the equilibrium was obtained.

Thioredoxin-like disulfide reductases fall in two categories: thioredoxins catalyzing disulfide reduction possess reducing active-site cysteine pairs with intrinsic redox potentials (*E*′_0_) around −270 mV, and members of the thioredoxin family catalyzing disulfide bond formation and isomerization exhibit much higher redox potentials in the range of −120 mV to −180 mV (24). To get first insights into the function of Precambrian thioredoxins, we determined the intrinsic redox potentials (*E*′_0_ values) of the purified ancestral variants (Fig. S2) by measuring their disulfide exchange equilibrium constants with *E. coli* thioredoxin (ecTrx) (*E*′_0_ = −270 mV) as reference redox protein (Fig. 2). In addition, we included human Trx (hTrx) as a modern mammalian thioredoxin that is evolutionarily distant from ecTrx in the analysis. Specifically, oxidized or reduced ecTrx was incubated for 14 h at pH 7.0 and 25 °C with different concentrations of the respective reduced or oxidized ancestral Trx variant. All reaction products (oxidized and reduced ecTrx and oxidized and reduced ancestral Trx variant) could be separated with single reversed-phase HPLC runs (Fig. 2). Peak areas were then converted to concentrations, from which the respective disulfide exchange equilibrium constants (*K*_eq_, see Equation 1 under “Experimental procedures”) were calculated. All *K*_eq_ values proved to be independent of the initial mixing ratios, demonstrating that equilibrium had been attained for all reactions (Fig. S2). The redox potentials of the ancestral thioredoxins were calculated from the *K*_eq_ values with the Nernst equation (see Equation 2 under “Experimental procedures”). Table 1 shows that all ancestral thioredoxins exhibited strongly reducing redox potentials that only differed from that of ecTrx by less than 20 mV, indicating that Trx functioned as a strong reducing agent in the cell throughout evolution, even before the oxygen catastrophe that occurred about 2.45 billion years ago (Table S1) (25).

**Table 1:** **Redox potentials and thermodynamic stabilities of ancestral thioredoxins**

Trx variant/sequence identity with ecTrx	Redox potential*^a^*		Stability*^a^*		
	*K*_eq_	*E*′_0_	*D*_1/2_ (ox)*^b^*	*D*_1/2_ (red)*^b^*	ΔΔ*G*_ox/red_*^c^*
		*mV*	*m GdmCl*	*m GdmCl*	*kJ mol*^−*1*^
ecTrx/100%		−270	2.21	1.43	11.2 ± 0.2
LGPCA/84.4%	1.24 ± 0.04	−267 ± 0.40	4.37	3.59	7.50 ± 0.5
LPBCA/58.7%	1.50 ± 0.03	−265 ± 0.30	4.23	3.54	10.5 ± 0.6
LBCA/57.8%	1.43 ± 0.04	−265 ± 0.30	3.86	3.11	11.1 ± 0.9
LACA/53.2%	1.59 ± 0.02	−264 ± 0.20	4.07	3.47	7.00 ± 0.5
AECA/53.2%	1.49 ± 0.06	−265 ± 0.50	3.93	3.23	8.50 ± 0.6
LECA/38.2%	3.78 ± 0.07	−253 ± 0.20	3.75	2.99	8.10 ± 0.3
LAFCA/36.4%	0.53 ± 0.01	−278 ± 0.30	2.79	1.85	13.1 ± 0.4
hTrx/30.7%	1.09 ± 0.01	−269 ± 0.10*^d^*	ND*^e^*	ND	ND

*^a^* The indicated errors correspond to the standard errors obtained from the respective fits. We, however, estimate that the true errors of all indicated parameters are about 5% when pipetting errors, and errors in the determination of protein concentration are taken into account.

*^b^* Midpoint of the GdmCl-dependent equilibrium unfolding/refolding transitions at pH 7.0 and 25 °C.

*^c^* Values were calculated at the GdmCl concentration corresponding to the mean value of the transition midpoints (*D*_1/2_) of the oxidized and reduced form.

*^d^* This value is very similar to the redox potential values of hTrx determined previously by Scotcher *et al.* (54) and Thurlow *et al.* (55) (−281 and −276 mV, respectively), but differs significantly from the value of −230 mV reported by Watson *et al.* (56). Possible reasons for this discrepancy were discussed in detail by Scotcher *et al.* (54).

*^e^* ND means not determined.

**Figure 3.**
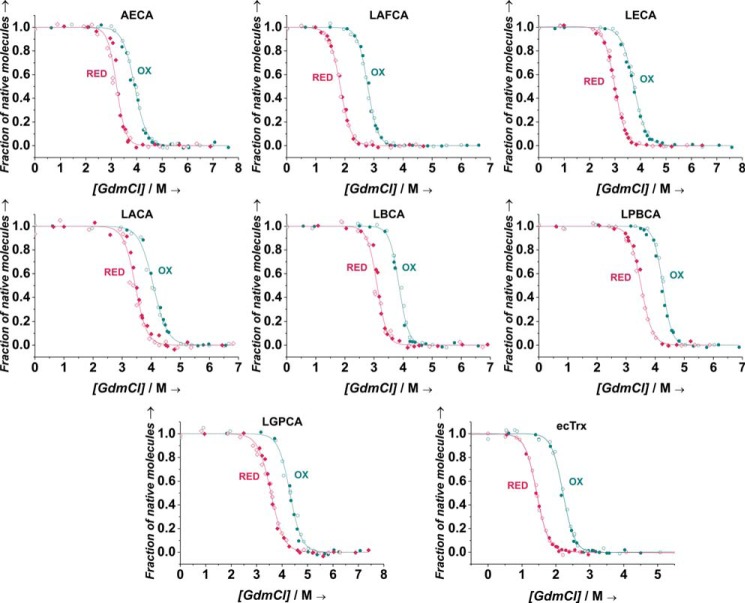
**GdmCl-dependent unfolding/refolding equilibria of oxidized (*blue*) and reduced (*red*) ancestral Trxs at pH 7.0 and 25 °C.** Unfolding (*open symbols*) and refolding (*closed symbols*) reactions were incubated for ≥24 h prior to the recording of the far-UV CD signal at 220 nm. Data for each unfolding/refolding equilibrium were fitted globally according to the two-state model of folding (Equation 3) and normalized (*solid lines*). The obtained thermodynamic stabilities of oxidized and reduced Trxs are summarized in Table 1 and Table S3.

Another hallmark of thioredoxins acting as reductases is the higher thermodynamic stability of their oxidized (disulfide) state relative to their reduced (dithiol) state, which thermodynamically drives substrate reduction based on a thermodynamic cycle that links the redox potential of thioredoxin with the free energies of folding of its oxidized and reduced form (26). Therefore, we determined the stabilities against unfolding of the oxidized and reduced forms of all Trx variants with guanidinium chloride (GdmCl)-induced unfolding/refolding transitions. Fig. 3 shows the corresponding unfolding/refolding transitions recorded via the decrease in the far-UV CD signal at 220 nm upon unfolding. All transitions were consistent with the two-state model of folding (see Equation 3 under “Experimental procedures”). As predicted from their reducing redox potentials, the oxidized forms of all variants indeed proved to be 7–13 kJ/mol more stable at pH 7.0 and 25 °C than the respective reduced forms (Table 1 and Table S3). In addition, extrapolation of the free energies of folding at pH 7.0 and 25 °C to zero denaturant revealed that the reduced ancestral thioredoxins were 4–25 kJ/mol more stable than reduced ecTrx and that all oxidized ancestral thioredoxins were 8–25 kJ/mol more stable than oxidized ecTrx (Table S3*A*). Comparison of the high thermodynamic stabilities at pH 7.0 and 25 °C of the ancestral thioredoxins with their high melting temperatures at pH 7.0 determined previously (15) revealed a similar order of stability, with ecTrx being the least and LPBCA the most stable variant (Table S3*B*).

### Reductase activity of the ancestral thioredoxins toward non-natural substrates

**Figure 4.**
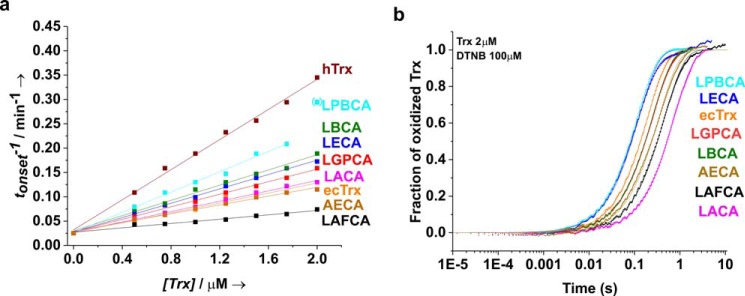
**Reactivity of ancestral thioredoxins as reductants of non-natural disulfide substrates at pH 7.0 and 25 °C.** *a,* activity of ancestral thioredoxins as catalysts of insulin (0.13 mm) reduction by DTT (1 mm). Reactions were followed by the increase in optical density at 650 nm, caused by aggregation of the reduced insulin B chain. The inverse time of aggregation onset (time required for an increase in OD_650_ of 0.05) depended linearly on catalyst concentration between 0.5 and 2.0 μm Trx. The slopes of the linear regressions were defined as the specific insulin reductase activity. The last LPBCA data point at 2.0 μm Trx (*in brackets*) is an outlier and was not included in the linear fit. *b*, reduction of DTNB by the reduced Trx variants, recorded with stopped-flow kinetics under pseudo-first–order conditions (100 μm DTNB, 2 μm Trx_red_) via the increase in TNB absorbance at 412 nm. Original data were fitted according to pseudo-first–order kinetics (see Table 2) and normalized (*solid lines*).

We analyzed the reactivity of the reduced ancestral thioredoxins with different disulfide substrates. First, we determined their activity as catalysts of the reduction of insulin disulfides by dithiothreitol (DTT) at pH 7.0 and 25 °C, which can be monitored via the increase in optical density at 650 nm (OD_650_) due to the aggregation of reduced insulin B chains (27). We established conditions under which the inverse time of aggregation onset (*t*_onset_^−1^), defined as the time required to reach an OD_650_ value of 0.05, depended linearly on Trx concentration. The slopes of the corresponding linear regressions therefore corresponded to the specific insulin reductase activities of the ancestral thioredoxins (Fig. 4*a*). Table 2 shows that all ancestral thioredoxins exhibited insulin reductase activity similar (within a factor of ∼2) to that of ecTrx. These data differ from a previously reported study at pH 5.0 in which the Trx variants LBCA, LACA, and AECA reduced disulfides in mechanically unfolded proteins about 1 order of magnitude faster than ecTrx (15). This could possibly be explained by a lower active-site cysteine p*K_a_* value in ancestral thioredoxins compared with ecTrx. Fig. 4*b* and Table 2 show that the ancestral thioredoxins also reduced 5,5-dithiobis-(2-nitrobenzoic acid) (DTNB, Ellman's reagent) (28) at pH 7.0 with rate constants (*k*_DTNB_) not differing more than 4-fold from that of ecTrx. As expected, the recorded *k*_DTNB_ values increased with increasing insulin reductase activity of the Trx variants (Fig. S4*a*).

**Table 2:** **Kinetic parameters of ancestral thioredoxins** Interaction of the ancestral thioredoxins with the non-natural substrates insulin and DTNB.

Trx variant/sequence identity with ecTrx	Specific insulin reductase activity*^a^*	Insulin reductase activity relative to ecTrx	*k*_DTNB_*^a^*	*k*_DTNB_ relative to *k*_DTNB_ of ecTrx
	*min*^−*1*^ μ*m*^−*1*^		*m*^−*1*^ *s*^−*1*^	
ecTrx/100%	5.12 ± 0.28·10^−2^	1	5.10 ± 0.04·10^4^	1
LGPCA/84.4%	6.45 ± 0.19·10^−2^	1.26	4.01 ± 0.04·10^4^	0.80
LPBCA/58.7%	1.04 ± 0.04·10^−1^	2.02	9.08 ± 0.08·10^4^	1.78
LBCA/57.8%	7.81 ± 0.31·10^−2^	1.53	4.16 ± 0.01·10^4^	0.82
LACA/53.2%	5.32 ± 0.13·10^−2^	1.04	1.30 ± 0.04·10^4^	0.25
AECA/53.2%	4.52 ± 0.17·10^−2^	0.88	2.52 ± 0.06·10^4^	0.49
LECA/38.2%	7.38 ± 0.16·10^−2^	1.44	8.21 ± 0.03·10^4^	1.61
LAFCA/36.4%	2.24 ± 0.15·10^−2^	0.44	2.13 ± 0.04·10^4^	0.42
hTrx/30.7%	1.55 ± 0.04·10^−1^	3.03	ND*^b^*	

*^a^* Indicated errors correspond to the standard errors obtained from the respective fits. We, however, estimate that the true errors of all indicated parameters are about 5% when pipetting errors and errors in determining protein concentration are taken into account.

*^b^* ND means not determined.

### Activity of the ancestral thioredoxins as catalysts of methionine sulfoxide reduction by NADPH in E. coli

**Figure 5.**
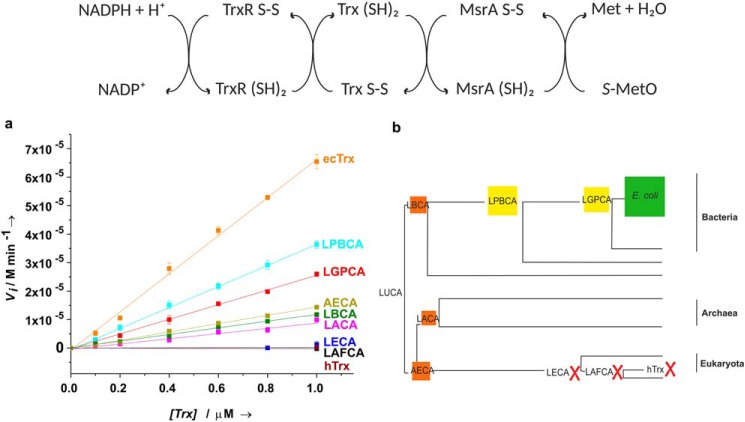
***In vitro* activity of ancestral thioredoxins as electron transfer catalysts in the reconstituted NADPH-dependent reduction of *S*-MetO at pH 7.0 and 25 °C, catalyzed by *E. coli* thioredoxin reductase (TrxR) and *E. coli* methionine sulfoxide reductase A (MsrA).** The *top panel* shows the complete scheme of the electron transport chain. *a*, specific activity of ancestral thioredoxins in NADPH-dependent *S*-MetO reduction. Initial velocities (*V_i_*), recorded via the decrease in NADPH absorbance, were measured as a function of Trx concentration. Initial concentrations (0.4 mm NADPH, 4.5 μm TrxR, 4.5 μm MsrA, 10 mm *S*-MetO) were chosen such that *V_i_* depended linearly on Trx concentration between 0.1 and 1 μm Trx. The slopes of the linear fits (*solid lines*) were defined as specific activity of the respective Trx variant in this assay. *b*, specific activity in NADPH-dependent *S*-MetO reduction of ancestral thioredoxins (proportional to the areas of the indicated squares) in the context of a phylogenetic tree. *Green square*, activity of ecTrx; *yellow squares*, Trx variants with a specific activity above 35% relative to that of ecTrx; *orange squares*, Trx variants with a specific activity of 10–35% relative to that of ecTrx; *red crosses*, Trx variants with less than 1% activity relative to that of ecTrx or no activity.

Next, we analyzed the function of the ancestral thioredoxins in the context of today's cellular *E. coli* environment *in vitro* and *in vivo*. Specifically, we selected *E. coli* peptide methionine sulfoxide reductase A (ecMsrA) from the group of known *in vivo* substrates of ecTrx. MsrA catalyzes the reduction of *S*-methionine sulfoxide (*S*-MetO) to methionine and possesses three essential active-site cysteines (Cys-51, Cys-198, and Cys-206). The catalytic cycle of ecMsrA involves (i) formation of a sulfenic acid intermediate at Cys-51 after *S*-MetO reduction to Met by reduced MsrA, (ii) the dissolution of the sulfenic acid intermediate by formation of a Cys-198–Cys-206 disulfide bond in MsrA, and (iii) the reduction of the Cys-198– Cys-206 disulfide by ecTrx that regenerates fully reduced MsrA (29–31). To quantify the specific activity of the ancestral thioredoxins as catalysts of MsrA reduction *in vitro*, we reconstituted the entire electron transfer pathway from NADPH via *E. coli* thioredoxin reductase (ecTrxR), Trx, and ecMsrA to *S*-MetO from purified components (Fig. 5*a*). We adjusted the experimental conditions so that initial velocities, recorded via the decrease in NADPH absorption at 340 nm, depended linearly on Trx concentration in the range of 0.1–1.0 μm Trx. Fig. 5*a* and Table 3 show that the resurrected bacterial Trx ancestors LGPCA and LPBCA maintained 39 and 55% of the specific activity of ecTrx and that the last bacterial, archaeal, and eukaryotic ancestors (LBCA, LACA, and AECA) still possessed 17, 14, and 22% of ecTrx activity, respectively. In contrast, all younger eukaryotic Trx ancestors (LECA and LAFCA) proved to possess less than 0.4% of ecTrx activity. Thus, the specific activities of the resurrected thioredoxins in the NADPH-dependent *S*-MetO reduction system of *E. coli* correlated well with the phylogenetic tree of evolution from the last bacterial common ancestor (LBCA) to *E. coli* (Fig. 5*b*) and their sequence identity with ecTrx (Fig. S4*b*).

**Table 3:** **Kinetic parameters of ancestral thioredoxins** Interaction of the ancestral thioredoxins with *E. coli* TrxR and *E. coli* MsrA. The indicated errors correspond to the standard errors obtained from the respective fits. We, however, estimate that the true errors of all indicated parameters are about 5% when pipetting errors and errors in determining protein concentration are taken into account.

	Catalytic activity of *S*-MetO reduction by NADPH (*V_i_*/[Trx])	Catalytic activity of *S*-MetO reduction by NADPH relative to ecTrx	Activity as substrate of TrxR (*k*_cat_/*K_m_*)	Activity as substrate of TrxR relative to ecTrx	Reaction with MsrA	
					*k*_1_*^a^*	*k*_2_*^b^*
	*min*^−*1*^		*m*^−*1*^ *s*^−*1*^		*m*^−*1*^ *s*^−*1*^	*s*^−*1*^
ecTrx	66.9 ± 1.6	1	8.81·10^6^	1	1.01 ± 0.01·10^6^	2.89 ± 0.01
LGPCA	25.8 ± 0.5	0.39	7.18·10^5^	0.081	3.69 ± 0.02·10^5^	1.65 ± 0.01
LPBCA	36.9 ± 0.7	0.55	7.60·10^6^	0.863	4.36 ± 0.01·10^5^	2.93 ± 0.01
LBCA	11.7 ± 0.2	0.17	1.17·10^5^	0.013	4.98 ± 0.01·10^5^	2.69 ± 0.01
LACA	9.13 ± 0.70	0.14	1.18·10^5^	0.013	5.18 ± 0.07·10^5^	1.54 ± 0.01
AECA	14.6 ± 0.6	0.22	1.30·10^5^	0.015	7.16 ± 0.06·10^5^	1.75 ± 0.01
LECA	0.29 ± 0.1	0.004	*^c^*		4.07 ± 0.04·10^4^	3.51 ± 0.02
LAFCA	*^c^*		*^c^*		4.50 ± 0.06·10^4^	3.47 ± 0.02
hTrx	0.22 ± 0.1	0.003	*^c^*		2.21 ± 0.04·10^5^	3.63 ± 0.01

*^a^* Rate constant of mixed disulfide formation is shown.

*^b^* Rate constant of mixed disulfide dissociation to reduced MsrA and oxidized Trx is shown.

*^c^* Activity was too low to be determined accurately.

### Efficiency of electron transfer between the ancestral thioredoxins and TrxR and MsrA from E. coli

**Figure 6.**
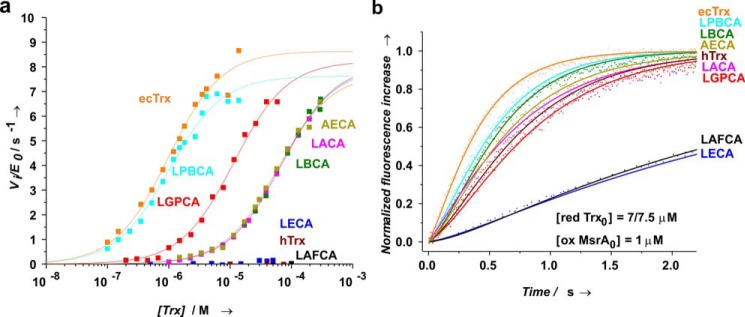
**Interaction between ancestral thioredoxins and *E. coli* TrxR (*a*) or *E. coli* MsrA (*b*) at pH 7.0 and 25 °C.** *a*, determination of the catalytic parameters of the reduction of ancestral thioredoxins by NADPH, catalyzed by *E. coli* TrxR. Reduced thioredoxins were converted back to their oxidized state *in situ* with excess DTNB, and the reactions were followed via the increase in absorbance at 412 nm due to formation of 2 eq of TNB per recycled, oxidized Trx. Initial velocities (*V_i_*) were plotted against Trx concentration. The deduced *K_m_* and *k*_cat_ values are summarized in Table 3. Initial concentrations were 0.8 mm NADPH, 3.3 mm DTNB, and 0.04 μm TrxR, and the initial Trx concentration was varied between 0.1 and 300 μm depending on the Trx variant. The Trx variants LECA, LAFCA, and hTrx were not recognized as substrates by *E. coli* TrxR. *b*, stopped-flow tyrosine/tryptophan fluorescence kinetics of reduction of *E. coli* MsrA by the ancestral thioredoxins at pH 7.0 and 25 °C. The initial concentration of oxidized *E. coli* MsrA (1.0 μm) was kept constant, and the initial concentrations of reduced ancestral thioredoxins were varied between 5.0 and 100 μm (pseudo-first–order conditions). As an example, the normalized fluorescence increase upon MsrA reduction at initial concentrations (7 or 7.5 μm) of reduced ancestral thioredoxins is shown. The global analysis of the kinetics revealed biphasic kinetics of MsrA reduction for all ancestral thioredoxins, which were particularly evident at low excess of reduced Trx over oxidized MsrA due to a lag phase in the fluorescence trace. All data were consistent with a consecutive mechanism, with a second-order reaction (rate constant *k*_1_) of mixed disulfide formation followed by a first-order decay (*k*_2_) of the mixed disulfide intermediate to reduced MsrA and oxidized Trx (*solid lines*). The deduced rate constants *k*_1_ and *k*_2_ are provided in Table 3 for all Trx variants investigated.

Two reasons could explain a lower or missing activity in NADPH-dependent *S*-MetO reduction of ancestral thioredoxins and hTrx, namely (i) less efficient reduction by *E. coli* TrxR than reduction of ecTrx and/or (ii) less efficient reduction of MsrA by ancestral thioredoxins compared with ecTrx. To address this question, we measured both reaction steps separately for all ancestral thioredoxins (Fig. 6 and Table 3). First, we determined the Michaelis-Menten parameters of the ecTrxR-catalyzed reduction of the ancestral thioredoxins by NADPH at saturation with NADPH and varying Trx concentrations. The results revealed a clear correlation between the activities of the ancestral thioredoxins in catalyzing the reduction of *S*-MetO by NADPH (Fig. 5) and their ability to interact with ecTrxR (Fig. 6*a* and Table 3). Specifically, the ancestral eukaryotic Trx variants LECA and LAFCA, which lacked catalytic activity in the reduction of *S*-MetO by NADPH, proved to be unable to interact with ecTrxR, whereas the two most active catalysts of NADPH-dependent *S*-MetO reduction, LPBCA and LGPCA (55 and 39% activity relative to ecTrx, respectively; Table 3), showed the highest *k*_cat_/*K_m_* values of all ancestral thioredoxins (86 and 9% relative to *k*_cat_/*K_m_* of ecTrx, Table 3), and the least active ancestral thioredoxins, LBCA, LACA, and AECA, showed more than 60-fold lower *k*_cat_/*K_m_* values than ecTrx (Table 3).

Second, we recorded the efficiency of reduction of the Cys-198–Cys-206 disulfide bond of ecMsrA by the ancestral thioredoxins with stopped-flow fluorescence kinetics, based on a strong increase in tryptophan fluorescence of MsrA that dominates over the decrease in Trx fluorescence upon oxidation (Fig. 6*b*). The reduction of MsrA by Trx is a two-step reaction. In the first step, the Cys-32 thiolate of Trx attacks the MsrA disulfide, generating a Trx–MsrA mixed disulfide (second-order rate constant *k*_1_). The second step is the attack of the mixed disulfide by Cys-35 of Trx and its dissociation to reduced MsrA and oxidized Trx (first-order rate constant *k*_2_). The kinetics of MsrA reduction by the ancestral thioredoxins, performed under pseudo-first–order conditions (1 μm oxidized MsrA and 5–100 μm reduced Trx), showed clearly-visible lag-phases as a consequence of formation of the mixed disulfide intermediate, which allowed us to extract the parameters *k*_1_ and *k*_2_ for all reactions (Figs. 6*b* and Fig. S5). Although all ancestral thioredoxins formed the mixed disulfide with MsrA slower (1.4–22-fold) than ecTrx, they still reacted rapidly to the mixed disulfide with MsrA, with rate constants *k*_1_ in the range of 10^4^–10^6^ m^−1^ s^−1^. In addition, all Trx–MsrA mixed disulfides spontaneously dissolved to oxidized Trx and reduced MsrA with essentially the same rates (within a factor of 2) as ecTrx (*k*_2_ = 1.8–3.6 s^−1^) (Table 3). Together, these results show that the ancestral thioredoxins were less active than ecTrx or inactive in catalyzing the NADPH-dependent reduction of *S*-MetO because they no longer interacted efficiently with ecTrxR, and not primarily because they did not efficiently reduce ecMsrA. This indicates that Trx and TrxR co-evolved during the molecular evolution of the reductive TrxR/Trx system and that Trx retained promiscuity toward its downstream substrates. This promiscuity is indeed not unexpected, as Trx has multiple downstream substrates besides MsrA (Fig. 1) that cannot be recognized individually with high specificity by Trx (32). In contrast, TrxR is the only redox enzyme from which Trx can receive electrons, predicting strong selective pressure on high specificity of the TrxR–Trx interaction.

### Complementation of Trx deficiency in E. coli under selective pressure by the ancestral thioredoxins

**Figure 7.**
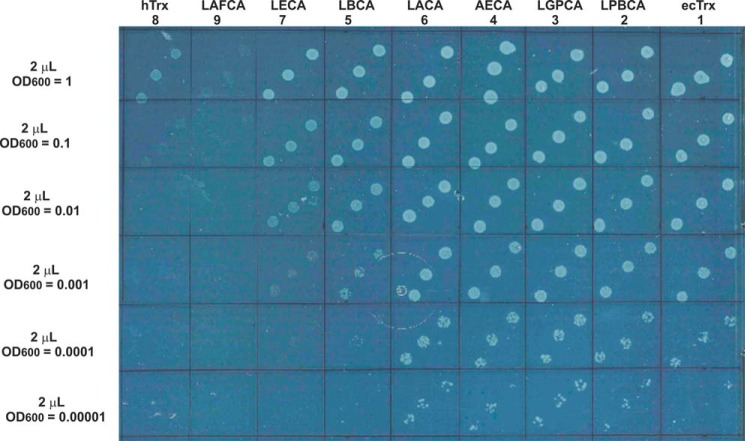
***In vivo* activity of ancestral thioredoxins in an *E. coli trxA/metE* deletion mutant under selective pressure in minimal medium with MetO as the sole source of methionine.** The *E. coli trxA/metE* deletion strain was transformed with expression plasmids for the respective Trx variants. All plasmids had an identical plasmid background and differed only in the genetic sequence of the respective *trx* gene. All thioredoxin genes were under the *trc* promoter/*lac* operator control. Cells were first grown in rich medium, harvested, washed, and then resuspended with minimal medium supplemented with MetO to OD_600_ of 1.0, 0.1. 0.01, 0.001, 0.0001, and 0.00001. Three 2-μl aliquots of each suspension were pipetted on an agar plate containing selective medium, ampicillin (120 μg/ml), kanamycin (50 μg/ml), and 1 mm IPTG and grown for 2 days at 25 °C. The *number below* each Trx variant corresponds to the ranking of its specific activity in catalyzing NADPH-dependent reduction of *S*-MetO in Table 3.

Next, we tested whether the enzymatic parameters of the ancestral thioredoxins recorded *in vitro* correlated with their ability to functionally replace Trx in today's *E. coli* context under selective pressure. As a selection system for functional Trx, we used a Δ*TrxA*Δ*MetE E. coli* strain deficient in ecTrx and methionine biosynthesis. This strain only grows in minimal medium supplemented with MetO as the sole source of methionine when complemented with an expression plasmid encoding active Trx. This selection system is based on the fact that the second thioredoxin in *E. coli*, Trx-2, does not contribute to MsrA reduction *in vivo* in the absence of oxidative stress (33). The Δ*TrxA*Δ*MetE* strain was transformed with the expression plasmids encoding the different ancestral Trx variants. The bacteria were first grown in rich medium to an optical density at 600 nm (OD_600_) of 1.0 and washed with selective medium, and aliquots of serial 10-fold dilutions down to OD_600_ = 0.00001 were applied to an agar plate with selective minimal medium supplemented with (*S*/*R*)-MetO. Fig. 7 shows that the Δ*TrxA*Δ*MetE* strains producing ecTrx or the Trx variants LGPCA, LPBCA, AECA, and LACA, which exhibited between 14 and 55% of the specific activity of ecTrx in NADPH-dependent reduction of *S*-MetO *in vitro* (Table 3), grew with the same efficiency and still formed colonies at the lowest dilution. Bacteria producing the Trx variant LBCA (17% activity relative to ecTrx) showed a small growth deficit; bacteria producing the almost inactive variants LECA and hTrx (0.4 and 0.3% *in vitro* activity relative to ecTrx, respectively) showed large growth deficits in this assay, and the Trx variant LAFCA (zero *in vitro* activity) completely failed to complement Trx deficiency (Table 3 and Fig. 7). Thus, the ability of the ancestral thioredoxins to complement Trx deficiency in the Δ*TrxA*Δ*MetE* strain in selective medium correlated very well with their specific activity in catalyzing the reduction of *S*-MetO by NADPH recorded *in vitro*.

As we could not distinguish between the efficiency of the most active ancestral Trx variants in complementing Trx deficiency via growth on agar plates (Fig. 7), we performed a true selective competition experiment in methionine-deficient liquid medium mimicking Trx evolution during the last 4 billion years. We inoculated the selective medium with a 1:1:1:1:1:1:1 mixture of the seven Δ*TrxA*Δ*MetE* strains transformed with the individual ancestral Trx variants, and we followed the population of the individual strains in this mixed culture during further bacterial growth. We expected that the strain expressing the ancestral Trx variant with the highest activity in catalyzing NADPH-dependent reduction of *S*-MetO in *E. coli* would eventually overgrow all other strains. We isolated 87–95 clones from the bacterial mixture when its optical density had increased 1000-fold, and we performed four cycles of a 10^3^-fold increase in cell density and sequencing. Table 4 shows that bacteria harboring two Trx variants LPBCA or LGPCA that had proven to be the most active in NADPH-dependent MetO reduction *in vitro* (Fig. 5) already became strongly enriched after a 10^3^-fold increase in cell density. In addition, only bacteria with these two variants could be detected after a 10^6^-fold increase in cell density, and the only bacteria that could be identified after a 10^12^-fold cell density increase were those harboring the most active ancestral Trx variant, LPBCA. This growth competition experiment is in excellent agreement with the specific activities of the ancestral thioredoxins recorded *in vitro* (Table 3), and also with the above-mentioned complementation experiments in the absence of selective pressure (Table S4) (23).

**Table 4:** **Enrichment of the most active ancestral thioredoxins in a growth competition experiment under selective pressure in MetO minimal medium**

Increase in OD_600_*^a^*	LPBCA	LGPCA	Others	Sequencing details
	%	%	%	
10^3^-Fold	77	21	2	91 clones sequenced
				70 clones: LPBCA
				19 clones: LGPCA
				1 clone: LACA
				1 clone: LECA
10^6^-Fold	94.5	5.5	0	95 clones sequenced
				90 clones: LPBCA
				5 clones: LGPCA
10^9^-Fold	99	1	0	87 clones sequenced
				86 clones: LPBCA
				1 clone: LGPCA
10^12^-Fold	100	0	0	95 clones sequenced
				95 clones: LPBCA

*^a^* Growth competition under selective pressure in minimal medium with MetO as the sole source of methionine was started with a 1:1:1:1:1:1:1 mixture of *E. coli* Δ*trxA*Δ*metE* cells transformed with expression plasmids for one of seven ancestral thioredoxin variants.

## Discussion

The strongly negative redox potentials and the higher stability of the oxidized relative to the reduced forms of the ancestral thioredoxins investigated in our study demonstrate that thioredoxins maintained their role as intracellular reductases throughout evolution. The negative redox potential of Trx guarantees that the electron transfer reactions from reduced Trx to its multiple oxidized substrates are always energetically favorable and do not become energetic or kinetic bottlenecks in the reaction cycle of Trx target enzymes. This could be confirmed with our selected natural Trx substrate MsrA, which was rapidly and quantitatively reduced by all ancestral thioredoxins when used as a stoichiometric reducing agent (Fig. 6*b* and Table 3).

**Figure 8.**
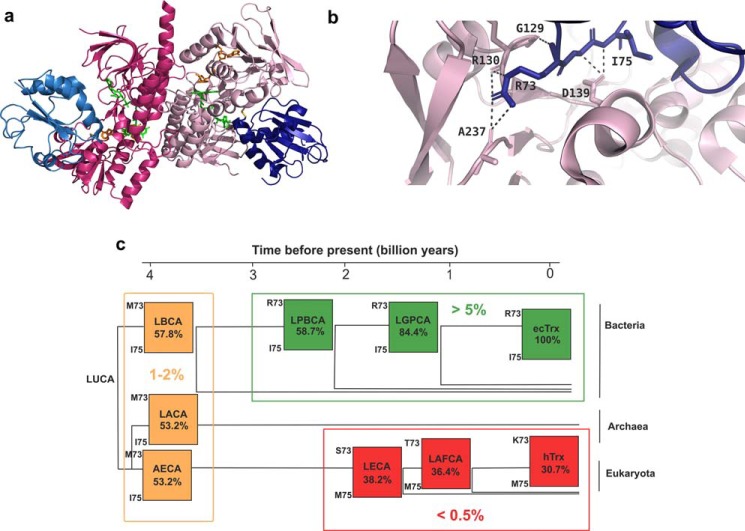
**Analysis of the interactions between ancestral thioredoxins and ecTrxR.** *a*, structure of the ecTrxR–ecTrx complex (PDB code 1F6M (39)). The two dimers of ecTrxR are colored in *two different shades of pink* and the two bound *E. coli* thioredoxins in *blue. Green,* FAD molecule bound to the FAD domain. *Orange,* 3-aminopyridine adenine dinucleotide phosphate, analogous of NADP^+^. *b*, detailed view of the interface between ecTrx and ecTrxR in the X-ray structure of the ecTrx–ecTrxR mixed disulfide complex, highlighting the specific intermolecular hydrogen bonds between ecTrx Arg-73 and ecTrxR Gly-129, Arg-130, and Ala-237, and between the main chain atoms of ecTrx Ile-75 and ecTrxR Asp-139. *c*, activity as a substrate of ecTrxR of ancestral Trxs (see Table 3) in the context of a phylogenetic tree. *Green squares*, ecTrx and Trx variants with TrxR substrate efficiency >5% relative to that of ecTrx; *orange squares*, Trx variants with a substrate efficiency of 1–2% relative to that of ecTrx; *red squares*, Trx variants with less than 0.5% substrate efficiency relative to ecTrx or no activity. The two residues corresponding to the Arg-73 and Ile-75 ecTrx in the respective ancestral Trx are shown at the *left corners of each square*. Below the name of each variant is reported its sequence identity relative to ecTrx.

Regarding the specificity and efficiency with which the ancestral thioredoxins interacted with today's physiological electron donor ecTrxR and different electron acceptor substrates, including the natural substrate ecMsrA and the non-natural substrate insulin, it is important to realize that TrxR is the single known protein from which Trx receives electrons (8, 9, 34), although it has numerous intracellular downstream substrates to which it transfers electrons. In fact, a proteomic analysis of Trx interactors in *E. coli* identified a total of 80 cellular proteins with which Trx interacts (32). A subset of these Trx downstream interactors is represented by redox enzymes to which Trx transfers electrons. This subset includes ribonucleotide reductase and multiple enzymes catalyzing the detoxification from reactive oxygen species, including MsrA, which only appeared after the oxygen catastrophe (35). The second subset of Trx interactors contains numerous proteins that are not redox-regulated, including transcription factors (32). Such a wide range of downstream *in vivo* substrates of Trx may indeed have been present from the beginning of evolution because ancestral enzymes are generally assumed to have been more promiscuous and less specific compared with modern enzymes (2, 36–38). Trx, in contrast to many other enzymes, appears to have retained a broad specificity for downstream substrates over four billion years of evolution. This, in turn, prevented Trx evolution toward high specificity for a single downstream substrate, in full agreement with our observation that all ancestral thioredoxins efficiently reduced the model substrates ecMsrA and even insulin (Table 2). In contrast to a large number of electron acceptor substrates, TrxR stayed as the only electron donor for Trx during evolution. This provides a plausible explanation for our hypothesis that Trx co-evolved with TrxR (Figs. 5 and 6). There are two classes of TrxR: low molecular weight thioredoxin reductases present in bacteria, archaea, fungi and plants, and high molecular weight enzymes present in higher eukaryotes and mammals (8, 34). Human TrxR, a member of the latter class, contains selenocysteine instead of a cysteine in its active site exchanging electrons with hTrx. The observation that hTrx does not interact with ecTrxR (Fig. 6*a*) thus also agrees with co-evolution of Trx with TrxR. We further analyzed the correlation between the activity of the ancestral thioredoxins as substrates of ecTrxR with their phylogenetic relationship to *E. coli* (Fig. 8 and Table 3) in the context of the solved X-ray structure of the mixed disulfide complex between ecTrx and ecTrxR (39). The analysis showed that two ecTrx residues in the loop preceding the invariant *cis*-Pro-76 (numbering according to ecTrx) form specific hydrogen bonds with ecTrxR (Fig. 8). Specifically, these residues are Arg-73 (side-chain atoms) and Ile-75 (main-chain atoms) that form hydrogen bonds with ecTrxR residues Gly-129, Arg-130, Ala-237, and Asp-139. Notably, the most efficient ancestral thioredoxins (LGPCA and LPBCA) possess the same residues as ecTrx at the corresponding positions (Arg-73 and Ile-75), whereas Arg-73 is replaced by Met-73 in the less efficient variants LBCA, LACA, and AECA, and both residues are replaced in the inactive variants LAFCA, LECA, and hTrx (Fig. 8). This is consistent with our hypothesis of co-evolution of Trx and TrxR and agrees with the finding that a *trans-*P76A variant of ecTrx, in which the loop segment 70–76 adopts a different conformation, no longer interacts with ecTrxR (40).

In conclusion, the present results, together with previous reports on the same set of ancestral thioredoxins, provided important insights into the functional evolution of thioredoxins in the cellular context. Another highly-interesting aspect of characterizing this set of ancestral enzymes is the possibility to predict their function in the context of the respective ancestral organisms and to predict the environmental conditions under which these organisms lived. The redox properties of the ancestral thioredoxins revealed their conserved function as reductases, indicating that the organisms living in the earliest time were already dependent on an efficient, NADPH-dependent defense system against oxidative stress. The most obvious reason for this is the electron transfer from TrxR to Trx, which is a prerequisite of DNA synthesis from ribonucleotide-building blocks. Moreover, the high thermodynamic stabilities of the ancestral thioredoxins (Fig. 3 and Table S3*A*) relative to that of ecTrx agree with the hypothesis that Precambrian organisms lived at high temperatures (17). Another hypothesis that could be tested with the collection of ancestral thioredoxins is that life started at low pH values (41, 42) and that the intracellular pH values in the earliest organisms may have been lower compared with that in modern organisms. The redox function of thioredoxins is based on fast disulfide-exchange chemistry, which is only possible if the attacking cysteine is present in the thiolate anion form (43–45). If the intracellular pH values of Precambrian organisms had indeed been more acidic, this predicts that the p*K_a_* of the nucleophilic, active-site cysteine thiol (Cys-32), which has an abnormal low value of 7.1 in ecTrx (46), is even lower in ancestral thioredoxins. Even though the Cys-32 p*K_a_* values of the ancestral thioredoxin have not yet been determined, the fact that the oldest ancestral Trx variants LBCA, AECA, and LACA reacted slower with DTNB at pH 7.0 than Trx from *E. coli* (intracellular pH 7.2–7.8 (47)) (Table 2 and Table S1) speaks against the hypothesis of a low intracellular pH in the oldest Precambrian organisms.

In summary, we showed that the evolution of Trx is an extraordinary example of molecular protein evolution, as Trx appears to have been both subject to evolution toward high specificity for its single electron donor TrxR and toward maintenance of promiscuity to its multiple downstream electron acceptors.

## Experimental procedures

### Reagents

Isopropyl β-d-thiogalactopyranoside (IPTG), DNase I, phenylmethylsulfonyl fluoride (PMSF), protease inhibitor EDTA-free mixture, DTT, bovine pancreas insulin, DTNB (Ellman's reagent), β-NADPH–reduced tetra(cyclohexylammonium) salt (NADPH), methionine sulfoxide (MetO), antibiotics, and all the chemical reagents used for buffer preparation were from Sigma-Aldrich Chemie GmbH. PD MiniTrap G-25 desalting columns and all other columns used for chromatography were from GE Healthcare.

### Data evaluation

Data evaluation was performed with Origin 9 (OriginLab).

### Expression plasmids

Synthetic genes encoding the seven ancestral Trx variants LAFCA, LGPCA, LECA, LACA, LBCA, LPBCA, and AECA (identical amino acid sequences to those published in Ref. 15) and hTrx were produced by GENEART and cloned into the expression plasmid pTrx (46) (*trc* promoter/*lac* operator control) via XbaI and HindIII. The *msrA* gene was cloned into the pDsbA2 (48) plasmid (*trc* promoter/*lac* operator control) via NdeI and BamHI after excision of the *dsbA* gene with the same restriction enzymes.

### Protein expression and purification and determination of protein concentrations

The thioredoxin variants LAFCA, LGPCA, LECA, LACA, LBCA, LPBCA, AECA, hTrx, ecTrx, and ecMsrA were expressed in *E. coli* BL21 (DE3). Bacteria were grown at 37 °C in LB medium containing ampicillin (120 μg/ml) until an OD_600_ of 0.6–0.8 was reached. Proteins were expressed for 3–6 h at 37 °C or 10–16 h at 25 °C after induction with 1 mm IPTG. After cell lysis (lysis buffer: 20 mm Tris-HCl, pH 7.5, for all proteins except for LACA (20 mm Tris-HCl, pH 9.0) and MsrA (20 mm Tris-HCl, pH 8.0), 50 μg/ml DNase I, 0.5 mm PMSF, protease inhibitor mix 1 tablet/50 ml), the soluble fractions of the cell extracts were loaded onto a Q-Sepharose column equilibrated with lysis buffer (without DNase I, PMSF, and protease inhibitor mix) and eluted with a linear gradient from 0 to 1 m NaCl (20 column volumes). Fractions containing the protein of interest were then mixed with ammonium sulfate to a final concentration of 1.5 m (except for MsrA, for which the final ammonium sulfate concentration was 1.2 m) and loaded onto a phenyl-Sepharose column equilibrated with 1.5/1.2 m ammonium sulfate, 20 mm MOPS-NaOH, pH 7.0. Proteins were eluted with a linear gradient from 1.5/1.2 to 0 m ammonium sulfate over 20 column volumes. Fractions containing the protein of interest were collected, concentrated to 13 ml, and loaded onto a Superdex 75 HiLoad 26/60 column equilibrated with 50 mm sodium phosphate, pH 7.0. Fractions containing pure protein were collected, flash-frozen in liquid nitrogen, and stored at −20 °C. For purification of hTrx and MsrA, all chromatography buffers additionally contained 5 mm DTT to guarantee purification of the fully reduced proteins. All other thioredoxin variants were obtained in the oxidized form, which was verified by ESI-mass spectrometry. TrxR was purified as described previously (49). Protein concentrations were determined via the specific protein absorption at 280 nm, using the following molar extinction coefficients: ecTrx 14,430 m^−1^ cm^−1^; LAFCA 7,960 m^−1^ cm^−1^; LGPCA 14,030 m^−1^ cm^−1^; LECA 8,300 m^−1^ cm^−1^; LACA 16,245 m^−1^ cm^−1^; LBCA 13,800 m^−1^ cm^−1^; LPBCA 14,640 m^−1^ cm^−1^; AECA 16,270 m^−1^ cm^−1^; hTrx_red_ 6,525 m^−1^ cm^−1^; TrxR (monomeric holoenzyme) 49,000 m^−1^ cm^−1^; and MsrA 36,120 m^−1^ cm^−1^. The final yields of purified thioredoxins were 20–70 mg/liter of bacterial culture and 45 mg/liter for MsrA.

### Redox equilibria

Redox potentials of ancestral Trx variants were calculated via their equilibrium constants with ecTrx (Equation 1), essentially as described in Ref. 50. Specifically, ecTrx_ox_ (10 μm) was incubated with equimolar amounts or 2–3-fold excess of the respective reduced Trx variant in 50 mm sodium phosphate, pH 7.0, 1 mm EDTA (final volume, 100 μl) and incubated for 14 h at 25 °C. The reduced forms of the Trx variants were obtained after incubation (18 h) with 5 mm DTT in 100 mm sodium phosphate, pH 8.0, and buffer exchange against 50 mm sodium phosphate, pH 7.0, 1 mm EDTA using PD MiniTrap G-25 columns. Redox equilibria were quenched by addition of 12 μl of 100% formic acid, and 50-μl samples were loaded onto a ZORBAX C8 reverse-phase HPLC column (300 Å, 5 μm, 4.6 × 250 mm) equilibrated with 35% acetonitrile in water, 0.1% TFA. The different redox forms were separated with an acetonitrile gradient (35–65% in water, 0.1% TFA, 30 ml) at a flow rate of 1 ml/min; HPLC separation was performed at 40 °C for samples containing the Trx variants LECA, LBCA, and AECA, all other separations were performed at 80 °C. Elution profiles were recorded via the protein absorbance at 220 nm, and peak areas were converted to concentrations assuming identical extinction coefficients for both redox forms of each Trx variant. Redox equilibrium constants (*K*_eq_) were calculated according to Equation 1, and the Nernst Equation 2 was used to calculate the redox potentials (*E*′_0_) of the Trx variants, with a value of −270 mV for *E*′_0_ of the ecTrx_ox_/ecTrx_red_ reference redox couple.

(Eq. 1) $$K_{\text{eq}}=\frac{\left[{\text{ecTrx}}_{\text{ox}}\right]\left[{\text{variant}}_{\text{red}}\right]}{\left[{\text{ecTrx}}_{\text{red}}\right]\left[{\text{variant}}_{\text{ox}}\right]}$$

(Eq. 2) $$E\prime_{0}=-270\;\text{mV}+\left(\frac{RT}{2F}\ln K_{\text{eq}}\right)$$

### GdmCl-dependent unfolding/refolding equilibria

Equilibrium unfolding and refolding transitions were recorded essentially as described previously (40). Briefly, oxidized or reduced Trx variants were transferred to water using PD MiniTrap G-25 columns and lyophilized. Lyophilized proteins were then dissolved either in 50 mm MOPS-NaOH, pH 7.0, 1 mm EDTA (unfolding experiments) or in 8.1 m GdmCl, 50 mm MOPS-NaOH, pH 7.0, 1 mm EDTA (refolding experiments). These stock solutions were then diluted 13-fold with 50 mm MOPS-NaOH, pH 7.0, 1 mm EDTA containing different concentrations of GdmCl. The final volume of each sample was 200 μl, and the final Trx concentration was 0.2 mg/ml in each sample. Samples of reduced Trx variants additionally contained 5 mm DTT. Samples were incubated for at least 2 days at 25 °C to reach the folding/unfolding equilibrium. Then, the CD signal of each sample at 220 nm was recorded for 1 min and averaged (JASCO-J710 CD spectrometer), and the exact GdmCl concentrations in the samples were measured via their refractive index (51). CD signals were plotted against GdmCl concentration, and the data were fitted according to the two-state model of protein folding and normalized according to Equation 3,

(Eq. 3) $$S_{\text{obs}}=\left(m_{n}\cdot \left[D\right]+S_{n}^{0}\right)+\left(\frac{m_{u}+\left[D\right]+S_{u}^{0}-m_{n}\cdot \left[D\right]-S_{n}^{0}}{\exp\left(\frac{\Delta G_{{\text{H}}_{2}\text{O}}^{0}+m_{\text{eq}}\cdot \left[D\right]}{RT}\right)+1}\right)$$

where *m_n_* and *m_u_* are the “slopes” of the pre- and post-transition baselines; [*D*] is the denaturant concentration; *S*^0^*_n_* and *S*^0^*_u_* are the signals of native and unfolded protein at zero denaturant; Δ*G*_H_2_O_^0^ is the free energy of folding at zero denaturant; and *m*_eq_ is the linear dependence of Δ*G*^0^ on [*D*].

### Insulin reductase assay

The activity of ancestral thioredoxins as catalysts of insulin reduction by DTT was measured essentially as described previously (27, 50). Solutions of thioredoxins (final concentrations in the assays, 0.5–2 μm) in 100 mm sodium phosphate, pH 7.0, 2 mm EDTA, 1 mm DTT were prepared in 96-well plates. The reactions were started by addition of bovine pancreas insulin to a final concentration of 130 μm, and aggregation of reduced insulin B chains 25 °C was followed for 60 min via the increase in optical density at 650 nm using a BioTek® Synergy^TM^ 3 plate reader instrument. The time of aggregation onset (*t*_onset_) was defined as the time required for reaching an OD_650_ value of 0.05. The specific insulin reductase activity of each Trx variant was obtained from the slope of the linear dependence of 1/*t*_onset_ on Trx concentration.

### Kinetics of DTNB reduction

Reduced Trx variants were prepared as described above and mixed with DTNB at 25 °C in 50 mm MOPS-NaOH, pH 7.0, 1 mm EDTA using 1:1 stopped-flow mixing. The final Trx concentration of 2 μm was kept constant in all experiments, and the final DTNB concentration varied between 10 and 60 μm. Formation of thionitrobenzoic acid (TNB) was followed via the increase in absorbance at 412 nm with an SX20 stopped-flow spectrophotometer (Applied Photophysics), using an extinction coefficient of ϵ_412 nm_ of 28,300 m^−1^ cm^−1^ for two TNB molecules formed per reduced DTNB. For each experiment, at least three absorbance traces were recorded, averaged, and fitted according to pseudo-first–order kinetics. The obtained pseudo-first–order rate constants (*k*_app_) were plotted against DTNB concentration, and second-order rate constants were obtained from the slope of the linear dependence of *k*_app_ on DTNB concentration.

### TrxR/Trx/MsrA-catalyzed reduction of MetO by NADPH

We first established that MetO from Sigma consisted of a 1:1 mixture of *S*-MetO and *R*-MetO. The specific rotation of the commercial mixture in aqueous solution was [α_*D*_^25^] = +12.9°, corresponding to 49.6% of *S*-MetO (the rotation angle of *S*-MetO in aqueous solution is [α_*D*_^25^] = +99° and that of *R*-MetO is [α_*D*_^25^] = −71.6°) (52). Consequently, the concentration of *S*-MetO in solutions containing (*S/R*)-MetO was half the concentration of (*S/R*)-MetO.

We then reconstituted the TrxR/Trx/MsrA-catalyzed electron transfer chain from NADPH to *S*-MetO in the *E. coli* cytoplasm from purified components *in vitro* and adjusted the conditions so that the recorded initial velocities depended linearly on a Trx concentration between 0.1 and 1.0 μm Trx. The reactions were performed at 25 °C in 50 mm MOPS-NaOH, pH 7.0, 0.4 mm EDTA. Specifically, NADPH, TrxR, MsrA, and the respective Trx variant were preincubated for 5 min before the reactions were started by addition of (*S/R*)-MetO and monitored for 1 min via the decrease in NADPH absorbance at 340 nm in a cuvette with a 0.3-cm path length. The final concentrations were as follows: 0.4 mm NADPH, 4.5 μm TrxR (monomer), 4.5 μm MsrA, and 5 mm *S*-MetO. The final Trx concentration was varied between 0.1 and 1.0 μm. The linear decrease in *A*_340_ with increasing reaction times was converted to initial velocity using a molar extinction coefficient of 6300 m^−1^ cm^−1^ for NADPH (53). The specific activity of each Trx variant in this assay was defined as the slope of the linear dependence of initial velocity on Trx concentration.

### Activity of the Trx variants as a substrate of E. coli TrxR

The activity of the Trx variants as a substrate of *E. coli* TrxR was quantified essentially as described (40). Reactions were performed at 25 °C in 50 mm MOPS-NaOH, pH 7.0, 0.4 mm EDTA. A mixture of TrxR and DTNB was preincubated at 25 °C while preparing solutions with different concentrations of the Trx variants in the above buffer in 96-well plates. The TrxR/DTNB mixture was added to the Trx solutions in the plates, and the reactions were started by addition of NADPH. The final concentrations were 0.84 mm NADPH, 0.04 μm TrxR, 3.3 mm DTNB, and 0.1–300 μm Trx. The reactions were monitored by following the increase in the absorbance at 412 nm due to TNB formation for 1 min in a BioTek® Synergy^TM^ 3 plate reader instrument. The initial velocities (calculated from an extinction coefficient of 28,300 m^−1^ cm^−1^ for the two TNB molecules formed per catalytic cycle) divided by the constant TrxR concentration (turnover numbers) were plotted against Trx concentration, and the data were evaluated according to Michaelis-Menten kinetics.

### Kinetics of MsrA reduction by the Trx variants

For production of oxidized *E. coli* MsrA (one intramolecular disulfide bond and one free thiol group), the DTT in the MsrA preparation was first removed by gel filtration (PD MiniTrap G-25 column). Reduced MsrA (100 μm) was then incubated for 15 min with 1 m eq of *S*-MetO in 50 mm MOPS-NaOH, pH 7, 0.4 mm EDTA at 25 °C, and free methionine was removed with a PD MiniTrap G-25 column equilibrated with the above-mentioned buffer. To prepare reduced thioredoxins, the thioredoxin variants were freshly reduced with DTT as described above and buffer-exchanged against 50 mm MOPS-NaOH, pH 7.0, 0.4 mm EDTA with PD MiniTrap G-25 columns. The reduction of oxidized *E. coli* MsrA by the reduced thioredoxins was recorded at 25 °C in 50 mm MOPS-NaOH, pH 7.0, 0.4 mm EDTA with an SX20 stopped-flow fluorescence spectrophotometer (Applied Photophysics) via the increase in the MsrA fluorescence above 300 nm upon reduction (excitation at 280 nm), which dominated over the decrease in Trx fluorescence upon Trx oxidation. The final MsrA concentration was 1 μm in all experiments. Final Trx concentrations were varied in the range of 5–100 μm. For each reaction, at least three fluorescence traces were recorded and averaged. Fluorescence kinetics were fitted according to a consecutive reaction mechanism (concentration-dependent formation of a mixed disulfide complex, followed by the unimolecular (concentration-independent) reaction of the mixed disulfide complex to reduced MsrA and oxidized Trx) and normalized.

### In vivo complementation of Trx deficiency

*E. coli* JFC318-MG1655Δ*TrxA*Δ*MetE*-KanR, kindly provided by J. F. Collet (De Duve Institute, Belgium), was first transformed with the expression plasmids for the respective Trx variants and plated on rich medium (LB) containing ampicillin (120 μg/ml) and kanamycin (50 μg/ml). Small liquid cultures (3 ml) in LB/ampicillin/kanamycin LB medium were then grown overnight at 37 °C. The bacteria were harvested by centrifugation and washed twice with “MetO minimal medium” (Met-deficient M9 minimal medium containing 1× M9 salt mixture, 2 mm MgCl_2_, 0.1 mm CaCl_2_, 0.4% glucose, amino acid mix without Pro, Tyr, and Met (0.05 g/liter each), 0.025 mm Pro, 0.025 mm Tyr, 10 mg/liter thiamine, 10 mg/liter biotin, 0.02 mg/ml (*S/R*)-MetO, 120 μg/ml ampicillin and 50 μg/ml kanamycin) and suspended in MetO minimal medium to an OD_600_ of 1.0. The suspensions were serially diluted (1:10 dilution steps) with MetO minimal medium; aliquots of 2 μl were spotted in triplicate on agar plates with MetO minimal medium supplied with 1 mm IPTG, and bacteria were grown for 48 h at 25 °C.

### Growth competition between trxA deficient E. coli cells expressing ancestral thioredoxins

KanR cells carrying expression plasmids for the ancestral thioredoxin variants. Cultures of *E. coli* JFC318-MG1655Δ*trxA*Δ*metE*-KanR transformed with the plasmids for expression of the Trx variants LGPCA, LPBCA, LBCA, LACA, AECA, LECA, or LAFCA were grown in rich medium (LB/ampicillin/kanamycin). Bacteria were harvested by centrifugation, washed twice with MetO minimal medium, and pellets resuspended in MetO minimal medium to identical cell densities of OD_600_ = 1.0. The seven cultures were mixed at a 1:1:1:1:1:1:1 ratio, and 0.25 ml of this mixture were used to inoculate 250 ml of MetO minimal medium supplemented with ampicillin and kanamycin. Cells were grown at 25 °C until an OD_600_ of 1.0 was reached, corresponding to a 1000-fold increase in cell density. From this culture, 250 μl were used to again inoculate 250 ml of MetO minimal medium for another 1000-fold increase in cell density to OD_600_ = 1.0. These cycles of 1000-fold increase in cell density were repeated four times. After each cycle, an aliquot of the culture with OD_600_ = 1.0 was plated on LB agar plates, from which 96 single colonies were picked. The *trx* genes from these 96 clones were then amplified by colony PCR and sequenced (Eurofins Genomics).

## Author contributions

S. N., M. R., and R. G. data curation; S. N. formal analysis; S. N. and R. J. R. investigation; S. N. and R. J. R. visualization; M. R. and R. G. conceptualization; M. R. and R. G. supervision; M. R. and R. G. validation; M. R. and R. G. writing-original draft; M. R. and R. G. writing-review and editing; R. G. funding acquisition; R. G. methodology; R. G. project administration.

## Supplementary Material

Supporting Information

## Footnotes

2 The abbreviations used are:

Trx: thioredoxin
TNB: thionitrobenzoic acid
PDB: Protein Data Bank
TrxR: thioredoxin reductase
PAPS: phosphoadenosine phosphosulfate
Gdm-Cl: guanidinium chloride
DTNB: 5,5-dithiobis-(2-nitrobenzoic acid)
hTrx: human Trx
ecTrx: *E. coli* Trx
IPTG: isopropyl β-d-thiogalactopyranoside
PMSF: phenylmethylsulfonyl fluoride
S-MetO: *S*-methionine sulfoxide
MetO: methionine sulfoxide
LBCA: Last Bacterial Common Ancestor
LACA: Last Archaeal Common Ancestor
AECA: Archaeal–Eukaryotic Common Ancestor
LECA: Last Eukaryotic Common Ancestor
LAFCA: Last Animalia and Fungi Common Ancestor
LPBCA: Last Common Ancestor of the Cyanobacterial, *Deinococcus*, and *Thermus* group
LGPCA: Last γ-Proteobacteria Common Ancestor.
