# Supplementary material for: Functional analyses of ancestral thioredoxins provide insights into their evolutionary history
Source: J Biol Chem. 2019 Jul 31;294(38):14105–18. doi: 10.1074/jbc.RA119.009718 (PMC6755812; doi:10.1074/jbc.RA119.009718)
Supplement: Supporting Information [file supp_294_38_14105__index.html]

Functional analyses of ancestral thioredoxins provide insights into their evolutionary history — Functional analysis of ancestral thioredoxins — Functional analyses of ancestral thioredoxins provide insights into their evolutionary history — Functional analyses of ancestral thioredoxins — Supporting Information 

# Functional analyses of ancestral thioredoxins provide insights into their evolutionary history

## Supporting Information

- Functional analyses of ancestral thioredoxins provide insights into their evolutionary history - Supporting Tables and Figures and related references.
