# Supplementary material for: Functional analyses of ancestral thioredoxins provide insights into their evolutionary history
Source: J Biol Chem. 2019 Jul 31;294(38):14105–18. doi: 10.1074/jbc.RA119.009718 (PMC6755812; doi:10.1074/jbc.RA119.009718)
Supplement: Supporting Information [file supp_RA119.009718_153409_2_supp_370494_pv7x7g.pdf]

Functional analyses of ancestral thioredoxins provide insights into their evolutionary history

Silvia Napolitano[1], Robin J. Reber[1], Marina Rubini[2] and Rudi Glockshuber[1]*

[1] Institute of Molecular Biology and Biophysics, Department of Biology, Swiss Federal Institute of Technology Zurich, Otto-Stern-Weg 5, CH-8093 Zurich, Switzerland
[2] University College Dublin, School of Chemistry, Belfield, Dublin 4, Ireland

Running title: *Functional analysis of ancestral thioredoxins*

*To whom correspondence should be addressed: Prof. Dr. R. Glockshuber: Department of Biology, Eidgenössische Technische Hochschule Zürich, Otto-Stern-Weg 5, CH-8093 Zurich, Switzerland; rudi@mol.biol.ethz.ch; Tel. +41-44-633 68 19

Keywords: molecular evolution, thioredoxin (Trx), thioredoxin reductase, redox biology, methionine sulfoxide reductase, oxidative stress, phylogenetic reconstruction, electron transfer, redox homeostasis

---

Table of contents:

1. ***Tables***
   - **1.1. *Table S1.*** Thioredoxin variants used in this study: abbreviations, age and sequence identity with *E. coli* thioredoxin (ecTrx)
   - **1.2. *Table S2.*** ESI mass spectra of purified, ancestral Trx variants (oxidized forms).
   - **1.3. *Table S3***
     - a) Thermodynamic stability of the oxidized and reduced forms of ancestral thioredoxins deduced from GdmCl-dependent unfolding/refolding equilibria at pH 7.0 and 25°C
     - b) Comparison between thermal stability and free energy of folding at pH 7.0 of the ancestral Trx variants
   - **1.4. *Table S4.*** Comparison of the ancestral thioredoxins in their ability to replace ecTrx as a catalyst of NADPH-dependent reduction of S-MetO in the presence of *E. coli* TrxR and *E. coli* MsrA *in vitro* and *in vivo*
2. ***Figures***
   - **2.1. *Figure S1***
   - **2.2. *Figure S2***
   - **2.3. *Figure S3***
   - **2.4. *Figure S4***
   - **2.5. *Figure S5***

3. ***References***

1. Tables

Table S1.

Thioredoxin variants used in this study: Abbreviations, age and sequence identity with *E. coli* thioredoxin (ecTrx)

**Abbreviation**	**Trx variant** [a]	**Sequence identity to ecTrx (%)**	**Age (years)** [b]
**LGPCA**	Last γ-proteobacterial common ancestor	84.4	~1.6 * 10^9
**LPBCA**	Last proteobacterial common ancestor	58.7	~ 2.5 * 10^9
**LBCA**	Last bacterial common ancestor	57.8	~ 4 * 10^9
**AECA**	Archaeal and eukaryotic common ancestor	53.2	~ 4 * 10^9
**LACA**	Last archaeal common ancestor	53.2	~ 4 * 10^9
**LECA**	Last eukaryotic common ancestor	38.2	1.6 *10^9
**LAFCA**	Last animalia and fungi common ancestor	36.4	~ 1.4 * 10^9
**hTrx**	Human thioredoxin	30.7	today

[a] Amino acid sequences of ancestral thioredoxins were identical to those provided in Figure 1 of reference[1].
[b] Calculated ages of last common ancestors are from reference[2].

***Table S2*.**

ESI mass spectra of purified, ancestral Trx variants (oxidized forms)

	Trx form without Met[a]		Uncleaved Trx form with Met[a]		
**Trx variant / sequence identity with ecTrx**	**Calculated mass (Da)**	**Measured mass (Da)**	**Calculated mass (Da)**	**Measured mass (Da)**	**Percentage of Met1 cleavage[a]**
**LGPCA / 84.4%**	11578.3	11578.5	11709.5	-	100%
**LPBCA / 58.7%**	11857.7	11857.5	11988.9	11988.5	86%
**LBCA / 57.8%**	11911.8	11912.0	12043.0	12043.0	79%
**LACA / 53.2%**	11885.0	11885.0	12016.2	12016.0	61%
**AECA / 53.2%**	11885.9	11885.5	12017.1	12016.5	82%
**LECA / 38.2%**	11900.8	11900.5	12032.0	12032.0	28%
**LAFCA / 36.4%**	11760.4	11759.5	11891.6	11891.0	15%
**hTrx / 30.7% [b]**	11606.3	11606.5	11735.5	11737.5	>50%

[a] The percentage of N-terminal Met (Met1) cleavage for the variants AECA, LPBCA, LAFCA and LECA was calculated from the ratio of the peak areas after reversed-phase HPLC separation of the protein with and without Met (Figure 2 and Figure S2), where the cleaved Trx form always eluted earlier than the more hydrophobic, non-cleaved form. For all other Trx variants, the fraction of methionine cleavage was calculated from the ratio of the relative peak intensities in the ESI mass spectra shown in Figure S3.
[b] hTrx was purified under reducing conditions.

Table S3.

(a) Thermodynamic stability of the oxidized and reduced forms of ancestral thioredoxins deduced from GdmCl-dependent unfolding/refolding equilibria at pH 7.0 and 25°C

	reduced form			oxidized form			
**Trx variant/ sequence identity with ecTrx**	$D_{1/2}$ (M)	m_{eq} (kJ mol^{-1} M^{-1})	$\Delta G^0_{H_2O}$ (kJ mol^{-1})	$D_{1/2}$ (M)	m_{eq} (kJ mol^{-1} M^{-1})	$\Delta G^0_{H_2O}$ (kJ mol^{-1})	$\Delta\Delta G^0_{ox/red}$ [a] (kJ mol^{-1})
**ecTrx / 100%**	1.43	14.3±0.8	–20.5±1.2	2.21	14.4±1.0	–31.9±2.3	11.2±0.2
**LGPCA / 84.4%**	3.59	9.6±0.4	–34.4±1.4	4.37	10.7±0.6	–46.2±2.8	7.5±0.5
**LPBCA / 58.7%**	3.54	12.4±0.6	–43.7±1.9	4.23	15.7±0.8	–66.8±3.4	10.5±0.6
**LBCA / 57.8%**	3.11	14.6±1.4	–45.3±4.4	3.86	14.9±0.8	–57.6±2.9	11.1±0.9
**AECA / 53.2%**	3.23	11.4±0.9	–35.8±2.7	3.93	10.4±0.4	–40.9±1.6	8.5±0.6
**LACA / 53.2%**	3.47	10.1±0.5	–41.3±1.9	4.07	12.3±0.9	–42.3±3.1	7.0±0.5
**LECA / 38.2%**	2.99	11.6±0.3	–34.7±0.9	3.75	10.1±0.3	–37.6±1.1	8.1±0.3
**LAFCA / 36.4%**	1.85	13.2±0.4	–24.3±0.7	2.79	14.4±0.4	–40.2±1.3	13.1±0.4

[a] To avoid errors of ΔG^0 extrapolation over a wide range of denaturant concentrations to zero denaturant, the stability difference between the oxidized and reduced form of each Trx variant ($\Delta\Delta G^0_{ox/red}$) was calculated at the GdmCl concentration corresponding to the mean value of the transition midpoints ($D_{1/2}$) of the oxidized and reduced form. $\Delta\Delta G^0_{ox/red}$ is defined such that $\Delta\Delta G^0_{ox/red}$ is positive when the oxidized form is more stable than the reduced form. Indicated errors correspond to errors obtained from the fits according to the two-state model of protein folding.

(b) Comparison between thermal stability and free energy of folding at pH 7.0 and 25°C of the ancestral Trx variants

**Stability parameter**	**Order of Stability**
**Thermal stability (T_m), oxidized Trx variants**[a]	ecTrx < LAFCA < LECA < LGPCA < LBCA, AECA, LACA < LPBCA
**$\Delta G^0_{H_2O}$ (25°C) oxidized Trx variants**[b]	ecTrx < LECA < LAFCA, AECA, LACA <LGPCA < LBCA < LPBCA

[a] Melting temperatures at pH 7.0 recorded in the absence of reducing agents[2].
[b] This work, data from Table S3A.

***Table S4*.**

Comparison of the ancestral thioredoxins in their ability to replace ecTrx as a catalyst of NADPH-dependent reduction of S-MetO in the presence of *E. coli* TrxR and *E. coli* MsrA *in vitro* and *in vivo*

Complementation assay	Order of complementation efficiency
NADPH-dependent MetO reduction *in vitro* at 25°C [a]	LPBCA > LGPCA > AECA > LBCA > LACA > LECA, LAFCA
Activity as a substrate of ecTrxR *in vitro* at 25°C [b]	LPBCA > LGPCA > AECA, LBCA, LACA > LECA, LAFCA
Growth on selective MetO medium agar plates at 25°C [c]	LPBCA, LGPCA, AECA, LACA > LBCA > LECA > LAFCA
Growth on selective liquid MetO medium at 25°C [d]	LPBCA > LGPCA >> AECA, LBCA, LACA, LECA, LAFCA
Growth of a ΔTrx1ΔTrx2 strain in rich medium at 37°C [e]	LPBCA > LGPCA > AECA > LACA > LECA > LBCA > LAFCA

[a] From Figure 5A; [b] From Figure 6A; [c] From Figure 7; [d] From Table 3; [e] From[3]

3. *Figures*

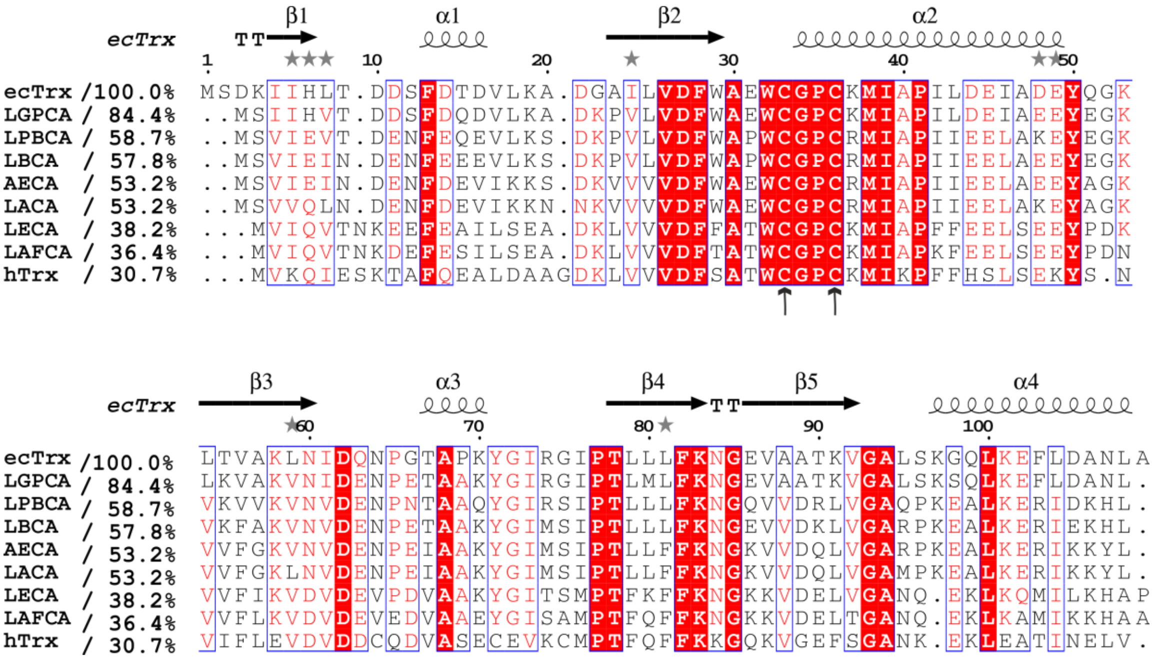

Figure S1**:** Structure-based sequence alignment of ecTrx with ancestral thioredoxins and hTrx using ESPript 3[4]. Residues are numbered according to ecTrx, and the sequences are sorted according to sequence identity with ecTrx (sequence identity is provided as percentage next to the variant name). The active site cysteines (C32 and C35) are marked by arrows. Fully conserved residues are in white on a red background. If the similarity score, according to physico-chemical properties, is above the threshold of 0.7, the column is framed in blue and the amino acids colored in red. Stars above the corresponding column indicate alternative conformations of the corresponding residue.

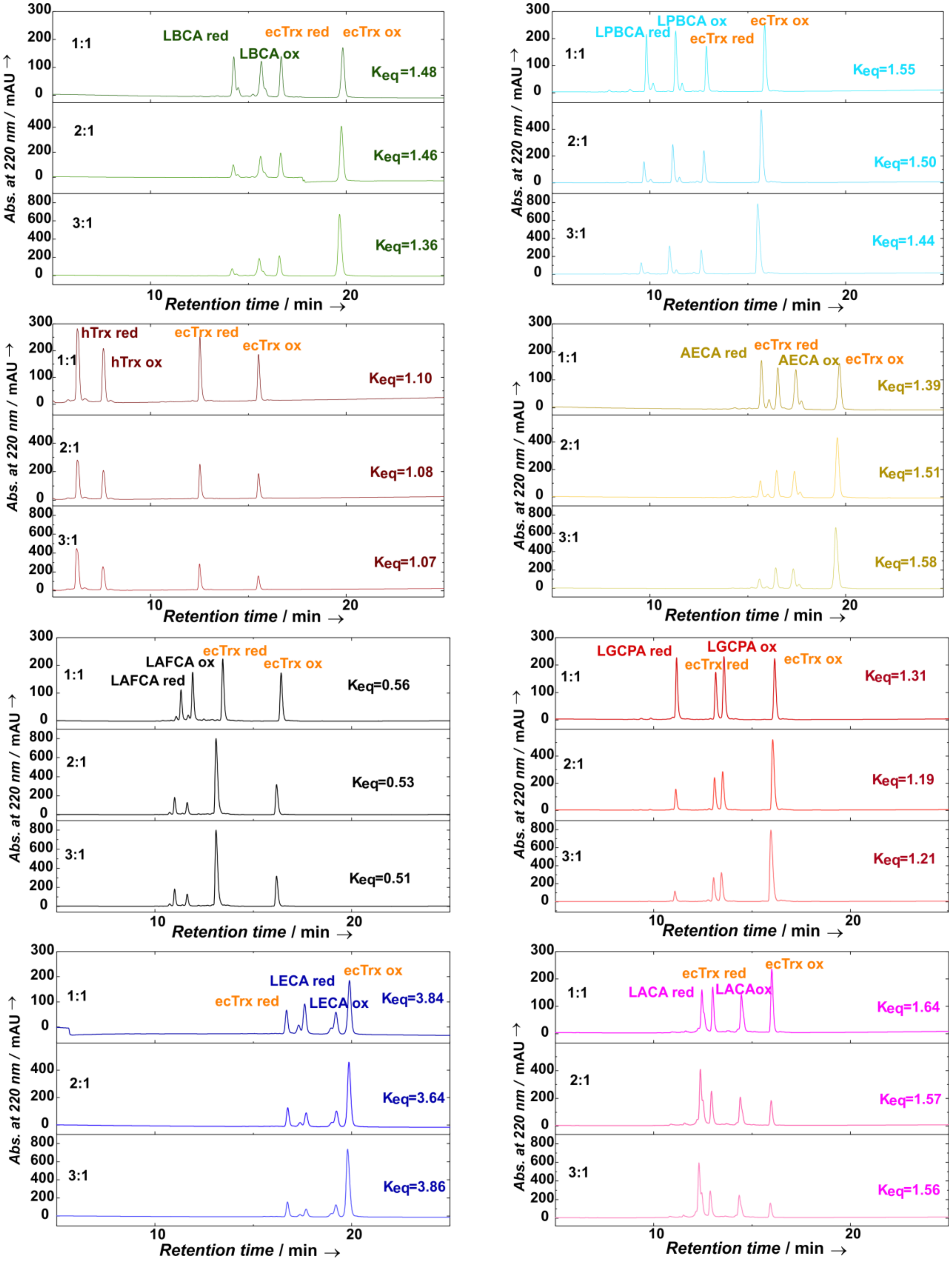

LBCA red
ecTrx red
ecTrx ox
LBCA ox
1:1
K_{eq}=1.48
2:1
Keq=1.46
3:1
Keq=1.36
LPBCA red
LPBCA ox
ecTrx red
ecTrx ox
Keq=1.55
Keq=1.50
Keq=1.44
hTrx red
hTrx ox
Keq=1.10
Keq=1.08
Keq=1.07
AECA red
AECA ox
Keq=1.39
Keq=1.51
Keq=1.58
LAFCA ox
LAFCA red
Keq=0.56
Keq=0.53
Keq=0.51
LGCPA red
LGCPA ox
Keq=1.31
Keq=1.19
Keq=1.21
LECA red
LECA ox
Keq=3.84
Keq=3.64
Keq=3.86
LACA red
LACAox
Keq=1.64
Keq=1.57
Keq=1.56
Abs. at 220 nm / mAU →
Retention time / min →

Figure S2. Redox equilibria between ecTrx and the individual ancestral thioredoxins at pH 7.0 and 25°C. Reduced ecTrx was mixed with the indicated molar equivalents of the oxidized form of the respective ancestral Trx variant. For each ancestral Trx variant, three different mixing ratios were used to determine the equilibrium constant (K_{eq}) of reduction by ecTrx. The reactions were quenched with acid, all redox forms were separated by reversed-phase HPLC, and peak areas were converted to concentrations, from which redox equilibrium constants (K_{eq}) were calculated (see Eq.1, Materials and Methods section). K_{eq} values were found to be independent of the mixing ratio between reduced ecTrx and oxidized ancestral Trx, showing that the redox equilibria were attained. The double peaks observed for both redox forms of the Trx variants AECA, LPBCA, LAFCA and LECA result from incomplete intracellular cleavage of the N-terminal methionine during expression of these variants (see Figure S3 and Table S2).

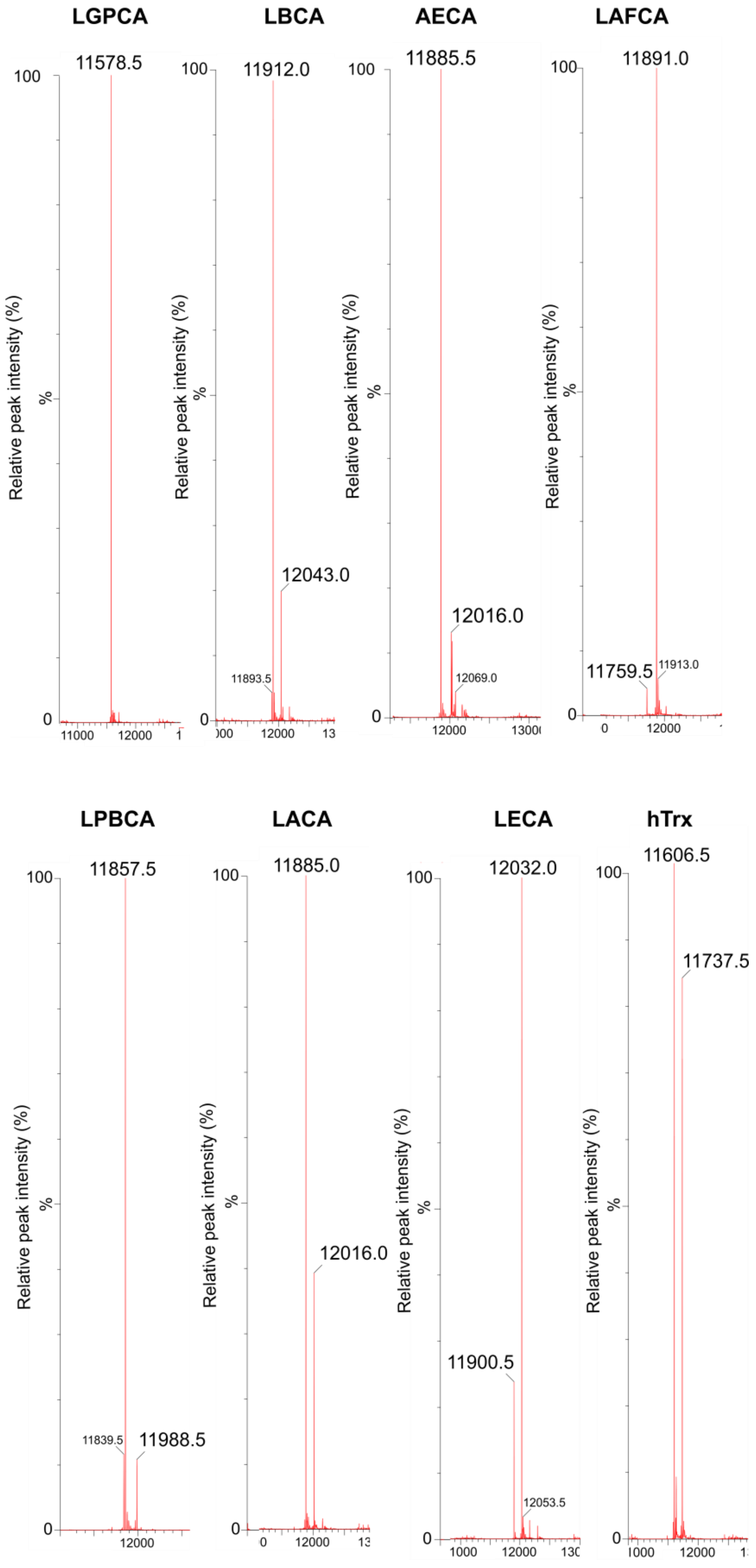

LGPCA
LBCA
AECA
LAFCA
11578.5
11912.0
12043.0
11893.5
11885.5
12016.0
12069.0
11891.0
11759.5
11913.0
Relative peak intensity (%)
LPBCA
LACA
LECA
hTrx
11857.5
11839.5
11988.5
11885.0
12016.0
12032.0
11900.5
12053.5
11606.5
11737.5

***Figure S3*:** ESI mass spectra of purified, ancestral thioredoxins (oxidized forms), showing that most ancestral thioredoxins were obtained as a mixture of the protein with and without the N-terminal methionine.

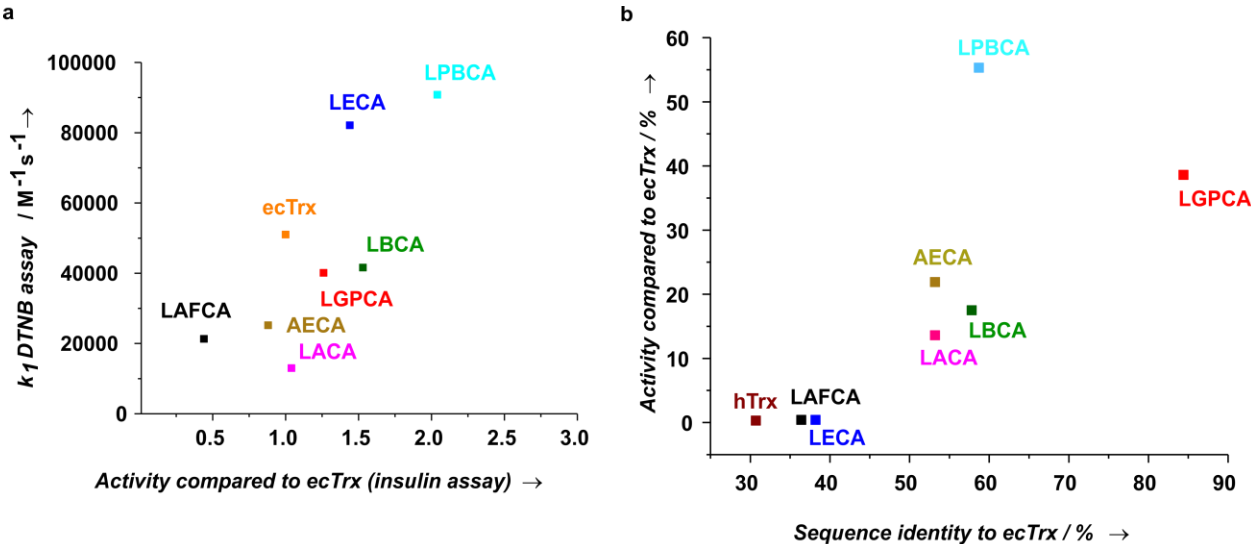

Figure S4. Correlation between the rate constants of DTNB reduction (k_{DNTB}) and the specific insulin reductase activities of ancestral thioredoxins relative to ecTrx (see Table 2A) (a), and correlation between the sequence identity of the ancestral thioredoxins with ecTrx and their specific activity as catalysts of NADPH-dependent reduction of S-MetO in the presence of *E. coli* TrxR and *E. coli* MsrA (b).

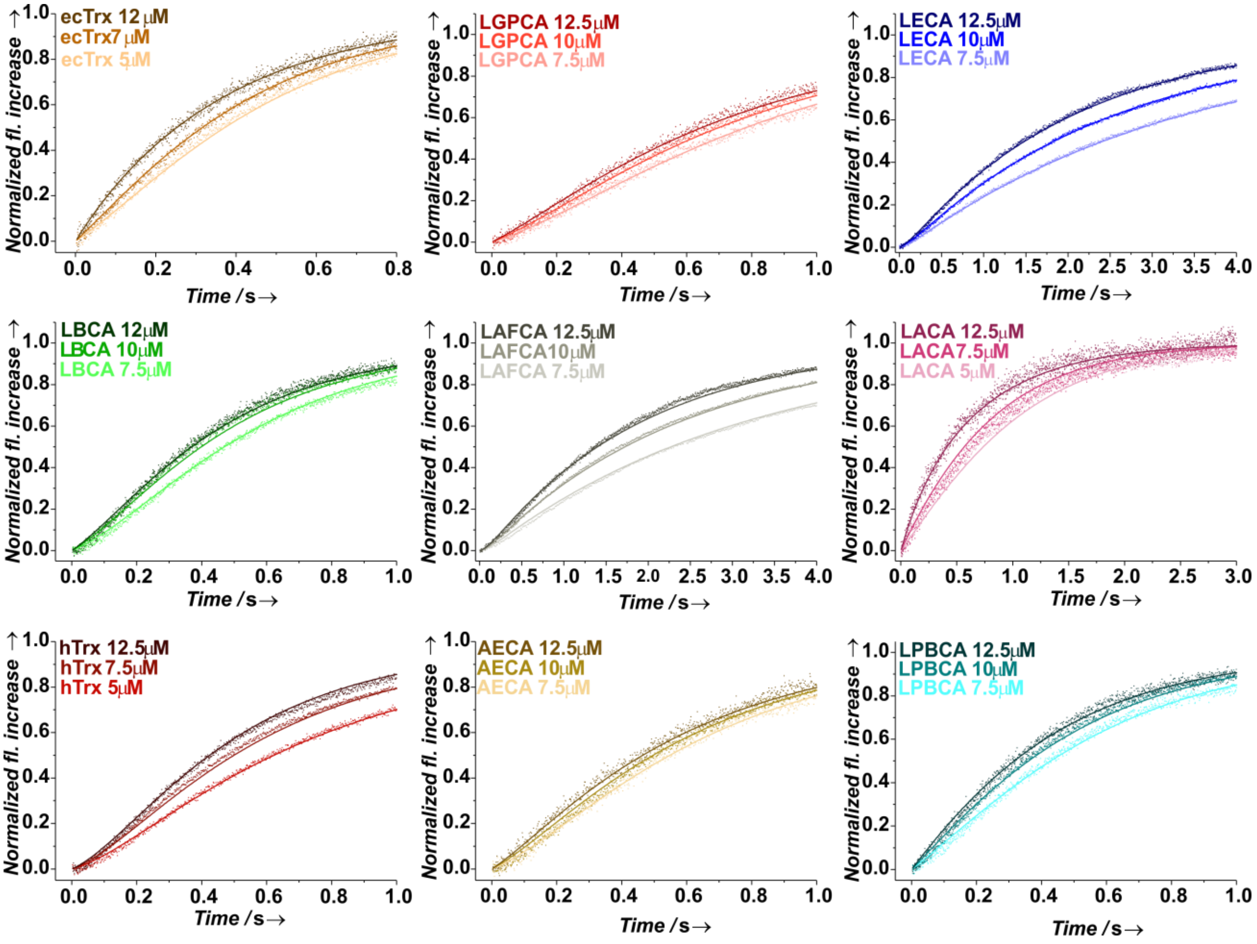

Figure S5. Stopped-flow fluorescence kinetics of the reduction of *E. coli* MsrA by the ancestral thioredoxins at pH 7.0 and 25°C, recorded under pseudo-first-order conditions at a constant MsrA concentration of 1.0 μM and different concentrations of excess, reduced thioredoxins. Three representative fluorescence traces are shown for the lowest concentrations of excess, reduced Trx where is visible the presence of the lag phase in all reactions. In each panel, the solid lines correspond to a global fit according to a consecutive mechanism, with a second-order reaction (rate constant k_1) of mixed disulfide formation followed by a first-order decay (k_2) of the mixed disulfide intermediate to reduced MsrA and oxidized Trx. The deduced rate constants k_1 and k_2 are provided in Table 2b for all Trx variants.

3. References

1. Ingles-Prieto, A. *et al.* Conservation of protein structure over four billion years. *Structure* **21**, 1690–1697 (2013).
2. Perez-Jimenez, R. *et al.* Single-molecule paleoenzymology probes the chemistry of resurrected enzymes. *Nat. Struct. Mol. Biol.* **18**, 592–596 (2011).
3. Delgado, A., Arco, R., Ibarra-Molero, B. & Sanchez-Ruiz, J. M. Using Resurrected Ancestral Proviral Proteins to Engineer Virus Resistance. *Cell Rep.* **19**, 1247–1256 (2017).
4. Robert, X. & Gouet, P. Deciphering key features in protein structures with the new ENDscript server. *Nucleic Acids Res.* **42**, W320–W324 (2014).
